# Using concept mapping in the development of the EU-PAD framework (EUropean-Physical Activity Determinants across the life course): a DEDIPAC-study

**DOI:** 10.1186/s12889-016-3800-8

**Published:** 2016-11-09

**Authors:** Giancarlo Condello, Fiona Chun Man Ling, Antonino Bianco, Sebastien Chastin, Greet Cardon, Donatella Ciarapica, Daniele Conte, Cristina Cortis, Marieke De Craemer, Andrea Di Blasio, Masar Gjaka, Sylvia Hansen, Michelle Holdsworth, Licia Iacoviello, Pascal Izzicupo, Lina Jaeschke, Liliana Leone, Livia Manoni, Cristina Menescardi, Silvia Migliaccio, Julie-Anne Nazare, Camille Perchoux, Caterina Pesce, Frank Pierik, Tobias Pischon, Angela Polito, Anna Puggina, Alessandra Sannella, Wolfgang Schlicht, Holger Schulz, Chantal Simon, Astrid Steinbrecher, Ciaran MacDonncha, Laura Capranica, on behalf of the DEDIPAC consortium

**Affiliations:** 1 Department of Movement, Human and Health Sciences, University of Rome Foro Italico, P.za Lauro de Bosis, 15, 00135 Rome, Italy; 2 Department of Physical Education and Sport Sciences, University of Limerick, Limerick, Ireland; 3 Institute of Sport, Exercise & Active Living, Victoria University, Melbourne, Australia; 4 Department of Psychology and Educational Science, University of Palermo, Palermo, Italy; 5 Institute for Applied Health Research, School of Health and Life Science, Glasgow Caledonian University, Glasgow, UK; 6 Department of Movement and Sports Sciences, Ghent University, Ghent, Belgium; 7 Council for Agricultural Research and Economics -Research Centre for Food and Nutrition, Rome, Italy; 8 Department of Human Sciences, Society, and Health, University of Cassino and Lazio Meridionale, Cassino, Italy; 9 Department of Medicine and Aging Sciences, ‘G. d’Annunzio’ University of Chieti-Pescara, Chieti, Italy; 10 Department for Sport and Exercise Sciences, University of Stuttgart, Stuttgart, Germany; 11 School of Health and Related Research (ScHARR)-Public Health Section, The University of Sheffield, Sheffield, UK; 12 Department of Epidemiology and Prevention. IRCCS Istituto Neurologico Mediterraneo: NEUROMED, Pozzilli, Italy; 13 Molecular Epidemiology Group, Max Delbrueck Center for Molecular Medicine in the Helmholtz Association (MDC), Berlin, Germany; 14 Counselling and Evaluation of Social and Health Policies - CEVAS, Rome, Italy; 15 Department of Applied Sciences in Physical Activity and Management, Catholic University of Valencia “San Vicente Mártir”, Valencia, Spain; 16 Centre de Recherche en Nutrition Humaine Rhône-Alpes, University of Lyon1, Lyon, France; 17 Department of Sustainable Urban Mobility & Safety, Nederlandse Organisatie voor Toegepast Natuurwetenschappelijk Onderzoek/Netherlands Organisation for Applied Scientific Research (TNO), Utrecht, The Netherlands; 18 Section of Hygiene - Institute of Public Health; Università Cattolica del Sacro Cuore, Fondazione Policlinico Universitario “Agostino Gemelli”, L.go F. Vito, 1-00168 Rome, Italy; 19 Institute of Epidemiology I, Helmholtz Zentrum München, German Research Center for Environmental Health, Munich/Neuherberg, Germany

**Keywords:** Factors, Active lifestyles, Youth, Adults, Older adults, Priority for research

## Abstract

**Background:**

A large proportion of European children, adults and older adults do not engage in sufficient physical activity (PA). Understanding individual and contextual factors associated with PA behaviours is essential for the identification and implementation of effective preventative environments, policies, and programmes that can promote an active lifestyle across life course and can potentially improve health. The current paper intends to provide 1) a multi-disciplinary, Pan-European and life course view of key determinants of PA behaviours and 2) a proposal of how these factors may cluster.

**Methods:**

After gathering a list of 183 potential PA behaviours-associated factors and a consensus meeting to unify/consolidate terminology, a concept mapping software was used to collate European experts’ views of 106 identified factors for youth (<19 years), adults (19–64 years), and older adults (≥65 years). The analysis evaluated common trends in the clustering of factors and the ratings of the distinct factors’ expected modifiability and population-level impact on PA behaviours across the life course. Priority for research was also assessed for each cluster.

**Results:**

The concept mapping resulted in six distinct clusters, broadly merged in two themes: 1) the ‘Person’, which included clusters ‘Intra-Personal Context and Wellbeing’ and ‘Family and Social Economic Status’ (42 % of all factors) and 2) the ‘Society’, which included the remaining four clusters ‘Policy and Provision’, ‘Cultural Context and Media’, ‘Social Support and Modelling’, and ‘Supportive Environment’ (58 % of all factors). Overall, 25 factors were rated as the most impactful on PA behaviours across the life course and being the most modifiable. They were mostly situated in the ‘Intra-Personal Context and Wellbeing’ cluster. Furthermore, 16 of them were rated as top priority for research.

**Conclusions:**

The current framework provides a preliminary overview of factors which may account for PA behaviour across the life course and are most relevant to the European community. These insights could potentially be a foundation for future Pan-European research on how these factors might interact with each other, and assist policy makers to identify appropriate interventions to maximize PA behaviours and thus the health of European citizens.

## Background

In line with the World Health Organization’s Global Status Report on Noncommunicable Diseases [1], the Council of the European Union [2] has recognised the value of physical activity (PA) for health and has provided recommendations on promoting health-enhancing physical activity (HEPA) across sectors and age groups. The policies of the European Union (EU) also strongly support grassroots and competitive sports [3], which are deeply intertwined with active lifestyles and represent an important opportunity to develop, transfer and/or implement regular PA practices [4], and to foster social inclusion, integration, and gender equality [5–8]. Unfortunately, the majority of European citizens do not engage in sufficient structured (e.g., physical exercise and sports) and/or unstructured (e.g., movements linked with daily life) PA, with a large proportion of children, adults, and older adults adopting inactive lifestyles [9–11]. To counteract the growing social and economic costs of lifestyle-related diseases, the European platform for action on diet, PA, and health aims to increase engagement in PA in the life course of citizens, to foster research for a better understanding of HEPA, and to boost and disseminate effective health policies for the promotion of environments and values supportive of an active lifestyle [5].

Whether or not individuals choose a healthy lifestyle is influenced by a number of inter-dependent and multilevel factors. Several theories and models have been proposed to facilitate the exploration of active lifestyle choice [12]. Recently, ecological perspectives have been proposed as an effective approach in combating current physical inactivity levels [13–17]. Such comprehensive models commonly include individual (e.g., biological, psychological, and behavioural aspects), interpersonal (e.g., relationships with parents, relatives, peers, and socio-cultural networks), environmental (e.g., access/availability of tools/services, and proximal/distal built/natural surroundings), and policy (e.g., organizational and governmental aspects) dimensions. Existing models provide a valuable overview but have not used a systematic methodology (e.g., concept mapping) to engage and analyse multi-disciplinary views, to specify the interrelations between the identified factors that might mediate or moderate PA behaviours, or to indicate how these factors may vary across the life course. To identify key factors that promote or inhibit PA behaviours, an agreed consensus framework, which contains sufficient detail to drive the future research agenda, is necessary. This agenda should focus on how these various factors interact with each other and how individual and population variation in these factors and in their interaction have a causal impact on behaviour and health.

The vehicle for the current research is the Thematic Area 2 of the DEterminants of DIet and Physical ACtivity Knowledge Hub (DEDIPAC-KH). To address the complex social and health phenomenon of healthy lifestyles behaviours in Europe, the European Commission endorsed a Joint Programming Initiative to increase research capacity across Member States to engage in a common research agenda [18]. Twelve Member States adopted this strategy and supported the DEDIPAC-KH to realise joint collaboration and harmonisation among different scientific disciplines, to expand knowledge, to develop new insights and solutions in the stated domains of behaviours, and to prepare the ground for building a coherent approach towards diet and PA behaviours research at European level [19]. Within the DEDIPAC-KH, a cross-disciplinary team coordinated and integrated collaborative research efforts to address the theoretical and practical challenges related to the determinants of PA behaviours and their changes across the life course. The partners recognised the need to identify priorities, to create a unified vision among stakeholders, and to guide future research in Europe. Such harmonisation is essential if meaningful research breakthroughs in the understanding of behaviour and lifestyle choice are to be made. In particular, in the current paper the terms determinants and factors are used interchangeable because they are both referring to the factors associated with PA behaviour. The identification of key factors or distinct clusters of factors, which are known to contribute to behaviour choice, as well as their level of modifiability and priority to research, will facilitate European and national policy makers in planning more effective behaviour enhancing public health policies [20].

According to the literature [21–26], concept mapping is deemed a valuable systematic methodology that involves a system-based approach to integrate ideas across multi-, inter-, trans-disciplinary, and professional knowledge in order to improve theory development as a sound basis for public health policies [20]. The concept mapping procedure requests participants to generate and structure statements and to identify relevant factors related to the question of interest (e.g., determinants of PA behaviours). The subsequent sorting and rating of suggested factors allows the identification of distinct clusters [27], which are represented in a two-dimensional concept map [28]. Clusters located close to each other carry a similar meaning, whereas distant ones are less related [29]. The involvement of a diversity of experts and disciplines is a core strength, which provides a comprehensive theoretical base to inform the concept mapping analysis [30]. This method of rating, clustering and visually mapping concepts by experts and stakeholders has been applied to create logic models to integrate practical knowledge with scientific knowledge for applied decision-making in public health [20, 31–36] and to gain insights into promising active living intervention strategies [37–39].

The primary aim of the current study was to develop, using a concept mapping approach, a EUropean Physical Activity Determinants (EU-PAD) framework to be indicative of the current understanding of PA determinants, which can underpin the future European research agenda and contribute to improving the active lifestyles of European citizens across the life course. The proposed characteristics of the framework were as follows: 1) a European and life-course view of key factors; 2) additional definition regarding the specific nature of the factors when compared to exiting models; and 3) propose how these factors may group into clusters. It is anticipated that the framework will provide significant guidance to future determinant research within Europe and will also provide a structure to increase collaboration and the harmonisation of research methodologies.

## Methods

According to the literature on defining and conceptualising complex public health systems with many interacting parts acting at different levels [22–26, 28, 30–35, 37–40], a structured consensus protocol has been developed based on concept mapping. In particular, this method combines qualitative opinions with multivariate statistical analysis to enable a synthesis of experts’ opinions to gather and organise views into a conceptual framework. In line with a parallel DEDIPAC-KH study on systems of sedentary behaviours [41], in the present study data collection, accomplished between December 2013 and December 2015, encompassed multi-method means, including paper forms, face-to-face interactions, and web-based platforms organized in four main phases (Table 1): (I) preparation (terminology, protocol and inclusion criteria of experts), (II) generation of statements, (III) structuring (sorting and rating), (IV) analysis and interpretation. In particular, the development of the EU-PAD framework benefited from the engagement of the members of the DEDIPAC-KH research team and involved also a panel of Pan-European multi-disciplinary experts in areas directly or indirectly related to PA and sport. Data analysis from each phase was necessary before progressing to the following one.

**Table 1:** Concept mapping stages, content, time frame and characteristics of participants

Stage	Content	Time Frame	Participants
Preparation	Terminology, Protocol and Inclusion Criteria of Experts	December 2013-December 2014	DEDIPAC-KH Research Team
Generation of Statements	Maps (brainstorming): 183 Factors	January-June 2015	
	Factors Sorting: 106 Factors		
	Pilot Sorting and Rating		
	Synthesis		
	Identification and Recruitment of EU Experts		
Structuring	Sorting and Rating of Factors	July-September 2015	EU Experts
Analysis and Interpretation	Preliminary Analysis and Interpretation (Delphi)	October-December 2015	DEDIPAC-KH Research Team
	Final Consensus		EU Experts

### Preparation (terminology, protocol and inclusion criteria of experts)

Considering that PA definitions often lack sufficient detail and present homonymous terms that lead to confusion and difficulty in communication [42, 43], the DEDIPAC-KH research team (consisting of 23 participants from five partner nations) deemed necessary to agree on a DEDIPAC-KH consensus on common nomenclature for PA. In this study, PA encompasses any bodily movement produced by skeletal muscles that results in energy expenditure, which may be unstructured and everyday life activity, exercise that includes prearranged, deliberate and repetitive activity [44–47], and grassroots sports and competitive sports [4].

The DEDIPAC-KH research team developed a protocol to articulate the goal of the present study (i.e., “the development of a framework indicative of the current understanding of PA determinants which can underpin the future European research agenda and contribute to improving the active lifestyles of European citizens across the life course”). Then, standard operating procedures [41] were provided to the members of the DEDIPAC-KH research team to individually establish in an open-ended and non-judgmental fashion an exhaustive list of all potential factors that could influence PA behaviours for youth (<19 years), adult (19–64 years), and older adult (≥65 years) populations, and to organize them into graphic representations to uncover the salient associations among factors and map their importance and modifiability.

To identify and recruit European experts, the DEDIPAC-KH research team conducted a focused search for multi-disciplinary specialists with particular attention to categories of European stakeholders having a relevant role in PA and sport. The following inclusion criteria were used: Experts affiliated with European organizations (President, Secretary, Manager of European institutions/organizations in HEPA, leisure and recreation, and sport; members of national sports departments; partners in relevant European co-funded PA projects), and experts affiliated with academic or research institutions. In particular, a snowball reputation-based sampling procedure was used to ensure an adequate recruitment of European scholars based on their expertise in PA research within the designated categories of the European Research Council Primary Panel Structure (e.g., Life Sciences, Social Sciences and Humanities, and Physical Sciences and Engineering). Thus, 373 European experts were identified.

According to the literature on online surveys for academic research [48, 49], a pre-notification email providing information on the development of the EU-PAD framework was prepared for the online recruitment of the identified European experts received. Participation in the task was deemed voluntary and participants could withdraw from the study at any time without providing any reason, and incomplete response would not be considered. Informed consent was assumed with subjects’ reply that they were willing to participate. Furthermore, follow-up contacts have been planned to increase response rates [48, 49], especially important for online surveys including >20 items as they demand long time from the respondent [48]. Considering that the response rate for e-mail surveys tend to be lower than that of traditional mail surveys [49, 50], and when representatives of organizations are involved [51] especially for time consuming responses [48], a response rate between 20 and 30 % was considered fair [52].

### Generation of statements

During a workshop, the DEDIPAC-KH research team analysed a list of 183 potential factors associated with PA behaviours by eliminating repetitions, rewording similar statements, and condensing highly specific statements into broader ones. Thus, a synthesis of 106 factors was identified (see numbered items in Table 2). Each factor was individually rated on a Likert-type scale from 1 (lowest value) to 5 (highest value) regarding its level of modifiability (‘To what extent is a factor modifiable at any point across the life course?’) and population-level effect (‘To what extent does a factor have an expected impact on PA behaviours at the youth/adult/older adult population-level?’) for the three life course stages. The internal consistency of factors was ascertained by means of reliability estimates, considering a Cronbach’s alpha coefficient of ≥0.7 acceptable for internal consistency [53]. Based on the outcomes of the above process, the synthesis of the 106 factors was approved for the next phase of the research.

**Table 2:** List of identified factors by cluster in ascending order

Factor #	Statement by cluster	Priority for Research	Modifiability	Population Level Effect			*p*
				Youth	Adult	Old Adult	
Cluster 1: Intra-Personal Context and Wellbeing							
3	Actual Body Mass Index	3.6 ± 0.7	3.6 ± 1.1	3.4 ± 1.0	3.8 ± 0.9	3.4 ± 1.0	0.032
4	Actual PA level	4.1 ± 0.6	4.1 ± 0.8	3.9 ± 1.0	4.1 ± 0.8	4.0 ± 1.0	n.s.
5	Addictions (Smoking Gambling Drugs)	3.0 ± 0.8	3.1 ± 1.1	2.7 ± 1.2^b^	3.2 ± 1.1^a^	2.9 ± 1.2	0.015
7	Age	2.4 ± 1.0	1.8 ± 1.4	3.0 ± 1.4^c^	3.2 ± 1.4	3.7 ± 1.3^a^	0.002
10	Beliefs/Values	3.3 ± 0.8	3.2 ± 1.0	3.3 ± 1.2	3.6 ± 1.1	3.6 ± 1.1	n.s.
11	Capability to Combine Sport and Education/Work Requirements (Dual Career)	3.1 ± 0.7	3.2 ± 1.0	3.4 ± 1.3^c^	3.8 ± 1.1^c^	1.7 ± 1.0^ab^	<0.001
14	Cognitive Function	3.0 ± 0.9	2.7 ± 1.1	3.0 ± 1.3^c^	3.1 ± 1.2^c^	3.9 ± 1.0^ab^	<0.001
15	Conscious Control of Automated Body Movements	3.0 ± 0.9	3.0 ± 1.1	2.9 ± 1.1	2.8 ± 1.2	3.2 ± 1.3	n.s.
23	Emotions	3.3 ± 0.9	3.1 ± 1.3	3.5 ± 1.0	3.4 ± 1.0	3.5 ± 1.1	n.s.
26	Fear of Injuries/Falling	3.1 ± 0.6	3.0 ± 0.9	2.3 ± 1.1^bc^	2.9 ± 0.9^ac^	4.6 ± 0.6^ab^	<0.001
27	Feeling of Inadequacy (Too Clumsy/Too Old)/Teasing	3.3 ± 0.7	3.1 ± 1.0	3.5 ± 1.1	3.2 ± 0.8^c^	3.6 ± 1.1^b^	0.024
31	Gender	2.1 ± 0.8	1.5 ± 1.0	2.8 ± 1.3	2.7 ± 1.2	2.7 ± 1.2	n.s.
33	Genetics/Talent	2.0 ± 0.7	1.5 ± 0.9	2.9 ± 1.2^bc^	2.4 ± 1.2^a^	2.2 ± 1.3^a^	<0.001
38	Health Status	3.7 ± 0.7	3.3 ± 0.9	3.4 ± 1.1^bc^	4.0 ± 0.9^ac^	4.4 ± 0.8^ab^	<0.001
39	Hormesis (Dose–response)/Training Response	2.7 ± 0.9	2.7 ± 1.2	2.6 ± 1.1	2.9 ± 1.0	2.6 ± 1.0	n.s.
41	Intentions/Attitudes	3.5 ± 0.8	3.4 ± 1.0	3.5 ± 1.0	3.8 ± 1.1	3.8 ± 1.1	n.s.
44	Job/Occupation-Related Energy Expenditure	2.8 ± 0.8	2.8 ± 1.2	2.2 ± 1.1^b^	3.7 ± 1.1^ac^	2.0 ± 1.1^ab^	<0.001
46	Level of Autonomy/Time Management	3.4 ± 0.7	3.4 ± 1.0	2.9 ± 1.0^bc^	3.9 ± 1.0^ac^	3.4 ± 1.1^ab^	<0.001
47	Life Satisfaction	3.4 ± 0.8	3.3 ± 0.9	2.9 ± 1.2^bc^	3.7 ± 1.0^a^	3.8 ± 1.0^a^	<0.001
57	Overweight/Obesity In Previous Years	2.6 ± 0.7	2.0 ± 1.1	3.1 ± 1.1^b^	3.5 ± 1.1^a^	3.3 ± 1.1	0.028
64	Past Exercise Behaviour/Experience	2.7 ± 0.8	1.9 ± 1.2	3.2 ± 1.2^c^	3.5 ± 1.1	3.8 ± 1.1^a^	0.003
65	Perceived Barriers	3.4 ± 0.7	3.2 ± 1.0	3.3 ± 1.1^c^	3.6 ± 1.0^c^	4.1 ± 0.9^ab^	<0.001
66	Perceived Benefits of PA	3.7 ± 0.7	3.7 ± 1.0	3.1 ± 1.2^bc^	3.9 ± 1.0^a^	4.0 ± 0.9^a^	<0.001
67	Perceived Fatigue/Adverse Physiological Response	3.3 ± 0.8	3.2 ± 1.0	3.0 ± 1.1^c^	3.4 ± 1.0§	3.9 ± 1.0^ab^	<0.001
68	Perceived Safe Environment	3.3 ± 0.7	3.1 ± 1.0	3.1 ± 1.2§	3.3 ± 0.9^c^	4.0 ± 0.9^ab^	<0.001
70	Perceived Stress/Life Stressors	3.1 ± 0.7	3.0 ± 1.0	2.8 ± 1.2^bc^	3.7 ± 0.9^ac^	3.3 ± 0.9^ab^	<0.001
71	Personal Goals/Outcome Expectancies/Achievement Orientation/Motivation	3.8 ± 0.7	3.8 ± 1.0	3.6 ± 1.2^a^	4.1 ± 1.0^a^	3.8 ± 1.0	0.026
72	Personality Traits	2.8 ± 1.0	2.3 ± 1.1	3.2 ± 1.2	3.4 ± 1.2	3.3 ± 1.3	n.s.
73	Physical Fitness Levels (Strength, Endurance, Coordination, Agility, Flexibility)	3.9 ± 0.7	3.9 ± 0.9	3.7 ± 1.0	3.7 ± 1.0^c^	4.1 ± 1.0^b^	0.022
76	Psychological Disorders (Depression, Eating Disorders, Emotional Symptoms)	3.1 ± 0.8	2.8 ± 1.0	3.0 ± 1.2^c^	3.4 ± 1.2	3.6 ± 1.0^a^	0.002
85	Self PA Monitoring	3.5 ± 0.7	3.6 ± 1.1	3.1 ± 1.2^b^	3.6 ± 0.8^ac^	3.2 ± 0.9^b^	0.002
86	Self Perceptions (Awareness, Confidence, Efficacy, Body Image, PA level)	3.6 ± 0.8	3.4 ± 1.0	3.5 ± 1.0	3.8 ± 1.0	3.7 ± 1.0	n.s.
87	Self-Regulatory Ability	3.2 ± 0.9	3.2 ± 1.2	3.0 ± 1.0^bc^	3.4 ± 1.1^a^	3.5 ± 0.9^a^	0.003
88	Sensation Seeking	2.6 ± 0.9	2.5 ± 1.8	3.0 ± 1.3^c^	2.9 ± 1.2^c^	2.2 ± 1.0^ab^	<0.001
89	Sleep Quality/Quantity	3.2 ± 0.9	3.2 ± 1.1	3.1 ± 1.2^c^	3.3 ± 1.1	3.6 ± 1.0^a^	0.012
98	Sub-Pathology/Pathology/Injuries/Pain/Rehabilitation	3.0 ± 0.8	2.6 ± 1.1	2.6 ± 1.2^bc^	3.2 ± 1.1^ac^	4.0 ± 1.1^ab^	<0.001
100	Time Availability	3.3 ± 0.7	3.2 ± 1.0	3.3 ± 1.0^bc^	4.2 ± 0.8^ac^	2.8 ± 1.1^ab^	<0.001
Cluster 2: Family and Socio-Economic Status							
22	Educational Level (Parents/Relatives)	2.6 ± 0.8	2.1 ± 1.2	3.4 ± 1.1^c^	3.2 ± 1.1	2.8 ± 1.3^a^	0.003
25	Ethnicity	1.9 ± 0.7	1.5 ± 1.0	2.4 ± 1.1	2.2 ± 1.0	2.4 ± 1.2	n.s.
51	Marital Status (for Children: Marital Status of Parents)	2.4 ± 0.8	2.3 ± 1.2	2.1 ± 1.0^bc^	2.8 ± 1.1^a^	2.7 ± 1.2^a^	<0.001
63	Parents/Relatives/Peers Body Mass Index	2.5 ± 0.8	2.3 ± 1.1	3.0 ± 1.1^c^	2.8 ± 1.1^c^	2.2 ± 1.1^ab^	<0.001
69	Perceived Social Role	3.0 ± 0.8	2.9 ± 1.0	3.0 ± 1.3	3.1 ± 0.9	3.0 ± 1.0	n.s.
81	Rewards (Encouragement/Support)	3.5 ± 0.8	3.7 ± 1.0	3.7 ± 1.2^bc^	3.2 ± 1.0^a^	3.1 ± 1.0^a^	<0.001
90	Social Competence/Role	3.0 ± 0.8	3.0 ± 1.0	2.9 ± 1.2	3.1 ± 1.0	3.1 ± 1.0	n.s.
91	Social Economic Status/Personal Income (for Children: Parents’ Income)/ Level of Education	2.9 ± 0.8	2.5 ± 1.0	3.4 ± 0.9	3.5 ± 0.9	3.3 ± 1.0	n.s.
Cluster 3: Policy and Provision							
1	Academic Training Programmes for Health Practitioners	3.2 ± 0.8	3.7 ± 1.1	2.7 ± 1.2	2.8 ± 1.1	2.9 ± 1.2	n.s.
8	Architecture and Urbanization (Availability/Access/Proximity of Elevators Escalators Facilities In Public Buildings)	3.0 ± 0.9	2.9 ± 1.2	2.8 ± 1.0^bc^	3.2 ± 1.1^a^	3.5 ± 1.1^a^	<0.001
9	Availability/Access/Proximity of PA Organized Sport Facilities Tools	3.6 ± 0.7	3.4 ± 1.0	3.9 ± 0.8	3.6 ± 0.9	3.7 ± 1.0	n.s.
12	City Planning	3.0 ± 0.9	3.0 ± 1.1	2.8 ± 1.1	3.1 ± 1.1	3.1 ± 1.1	n.s.
13	City/Nation Density	2.0 ± 0.8	1.8 ± 0.9	2.1 ± 1.1	2.1 ± 1.0	2.2 ± 1.1	n.s.
16	Corporate Social Responsibilities	2.6 ± 0.8	2.8 ± 1.0	2.1 ± 1.0^b^	2.7 ± 1.1^a^	2.3 ± 1.1	0.004
17	Corporate Social Responsibility Interventions	2.8 ± 0.7	3.0 ± 1.0	2.3 ± 1.2^b^	2.9 ± 1.1^ac^	2.3 ± 1.2^b^	0.001
20	Distance to School/Work/Destination	3.0 ± 0.7	2.7 ± 1.0	3.5 ± 0.9^c^	3.5 ± 1.1^c^	2.8 ± 1.4^ab^	<0.001
21	Education Policies	2.9 ± 0.9	3.2 ± 1.1	3.6 ± 1.1^bc^	2.3 ± 1.1^a^	2.1 ± 1.1^a^	<0.001
24	Environmental Policies	3.0 ± 0.9	3.3 ± 1.1	2.6 ± 1.1	2.8 ± 1.1	2.7 ± 1.0	n.s.
28	Financial Measures and Regulation for PA and Sport	3.0 ± 0.8	3.0 ± 1.1	2.9 ± 1.0	3.0 ± 1.0	2.7 ± 1.1	n.s.
29	Fiscal Advantages For Sport Clubs/PA Services	2.9 ± 0.9	3.0 ± 1.1	2.8 ± 1.1	2.9 ± 1.2	2.5 ± 1.1	n.s.
30	Funding for Sport Federation	2.7 ± 0.8	3.0 ± 1.0	2.7 ± 1.0^c^	2.5 ± 1.0^c^	2.1 ± 1.0^ab^	<0.001
37	Health Education	3.5 ± 0.7	3.7 ± 1.1	3.3 ± 1.2	3.3 ± 1.0	3.3 ± 1.0	n.s.
40	Indoor Condition (Air Conditioning)	2.8 ± 0.8	3.1 ± 1.2	2.1 ± 0.9^c^	2.4 ± 0.8	2.8 ± 1.0^a^	<0.001
45	Leisure Activity Subsidy	3.0 ± 0.8	2.9 ± 1.1	3.0 ± 1.0	3.0 ± 1.0	3.0 ± 1.1	n.s.
48	Lobbying	2.4 ± 0.9	2.8 ± 1.3	1.9 ± 1.0	2.3 ± 1.1	2.0 ± 1.0	n.s.
50	Mandatory PA in Community/Schools	3.1 ± 0.8	3.4 ± 1.1	4.0 ± 1.0^bc^	2.4 ± 1.1^a^	2.3 ± 1.3^a^	<0.001
53	Media and Advertising Regulation by Public Authorities Corporate Social Responsibility Programmes	3.0 ± 0.8	3.2 ± 1.1	2.8 ± 1.2	2.8 ± 1.1	2.5 ± 1.0	n.s.
54	Mobility Policy	2.8 ± 0.9	3.0 ± 1.2	2.4 ± 1.0^c^	2.6 ± 1.1^c^	3.1 ± 1.2^ab^	<0.001
56	Outdoor Condition (Pollution and Weather-Season)	2.6 ± 0.6	2.3 ± 1.1	2.8 ± 1.0^c^	3.0 ± 0.9^c^	3.4 ± 1.0^ab^	<0.001
58	PA and Sport Organizations Advocacy	3.1 ± 0.9	3.2 ± 1.1	3.1 ± 1.1	2.9 ± 1.1	2.8 ± 1.2	n.s.
59	PA Education (at School/Work)/Knowledge of Effects of PA	3.4 ± 0.9	3.6 ± 1.1	3.7 ± 1.1^bc^	3.2 ± 1.3^a^	2.8 ± 1.1^a^	<0.001
60	PA Programs in School/Office/Community	3.6 ± 0.8	3.6 ± 1.0	4.1 ± 1.0^bc^	3.4 ± 1.1^a^	3.4 ± 1.2^a^	<0.001
61	PA Programs/Plans	3.6 ± 0.8	3.6 ± 1.1	3.8 ± 1.0	3.5 ± 1.1	3.6 ± 1.1	n.s.
77	Public Health	3.0 ± 0.8	2.9 ± 1.1	2.5 ± 1.1^bc^	3.1 ± 1.1^a^	3.4 ± 1.1^a^	<0.001
78	Public Organized Sport Events/PA Activities (Field Trips)	3.4 ± 0.7	3.6 ± 1.0	3.4 ± 1.0	3.0 ± 1.0	3.3 ± 1.1	n.s.
79	Public Transport Policies	3.0 ± 0.9	3.0 ± 1.1	2.7 ± 1.0	2.9 ± 1.1	3.2 ± 1.0	n.s.
80	Public Transport System	3.1 ± 0.8	3.0 ± 1.1	3.1 ± 0.9	3.0 ± 1.1^c^	3.4 ± 1.0^b^	0.034
82	Rights of Citizenship	1.9 ± 0.8	2.1 ± 1.1	1.7 ± 0.9	1.9 ± 0.9	1.7 ± 0.9	n.s.
83	School/Office Hours	2.7 ± 0.7	2.5 ± 1.0	3.3 ± 1.1^bc^	3.8 ± 0.9^ac^	1.7 ± 0.9^ab^	<0.001
84	School/Office Space	2.7 ± 0.8	2.7 ± 1.1	3.5 ± 1.1^c^	3.2 ± 1.2^c^	1.6 ± 0.8^ab^	<0.001
96	Sport Science Research	3.1 ± 0.9	3.5 ± 1.2	2.6 ± 1.1	2.7 ± 1.1	2.5 ± 1.1	n.s.
97	Sports Facilities	3.4 ± 0.7	3.3 ± 1.1	3.7 ± 0.9^c^	3.7 ± 0.8^c^	3.2 ± 0.9^ab^	0.001
102	Traffic	2.7 ± 0.9	2.6 ± 1.1	2.7 ± 1.1	2.8 ± 1.0	2.9 ± 1.2	n.s.
103	Traffic Policies	2.8 ± 0.9	2.9 ± 1.2	2.5 ± 1.1	2.8 ± 1.0	2.8 ± 1.2	n.s.
104	Transport Policies	2.9 ± 0.9	3.0 ± 1.2	2.6 ± 1.1^c^	2.8 ± 1.1	3.1 ± 1.2^a^	0.014
106	Urban Planning Policies	3.0 ± 1.0	3.0 ± 1.2	2.8 ± 1.1	2.9 ± 1.2	3.0 ± 1.2	n.s.
Cluster 4: Cultural Context and Media							
6	Advertisement	3.1 ± 0.9	3.3 ± 1.2	3.1 ± 1.1^c^	2.8 ± 1.0	2.6 ± 1.1^a^	0.004
18	Cultural Climate	2.6 ± 0.8	2.4 ± 1.0	2.8 ± 1.1	2.9 ± 1.1	2.9 ± 1.2	n.s.
19	Cyber Space	2.7 ± 0.9	3.0 ± 1.3	3.3 ± 1.4^bc^	2.5 ± 1.1^ac^	1.8 ± 0.9^ab^	<0.001
32	Gender Equality	2.6 ± 0.8	2.6 ± 1.1	2.7 ± 1.3	2.8 ± 1.2^c^	2.3 ± 1.0^b^	0.040
35	Group Activity (Outdoor/Indoor)	3.6 ± 0.7	3.5 ± 0.9	3.8 ± 0.8^b^	3.5 ± 0.8^a^	3.8 ± 0.9	0.016
42	Internet Availability	3.1 ± 0.9	3.4 ± 1.2	3.3 ± 1.2^bc^	2.7 ± 1.2^ac^	2.1 ± 1.0^ab^	<0.001
49	Local/National/Traditions Identity	2.3 ± 0.9	2.0 ± 1.0	2.6 ± 1.2	2.5 ± 1.1	2.6 ± 1.1	n.s.
52	Media	3.2 ± 0.8	3.3 ± 1.1	3.4 ± 1.1^c^	3.0 ± 1.1	2.7 ± 1.0^a^	0.001
94	Social Media	3.2 ± 0.9	3.5 ± 1.2	3.7 ± 1.2^bc^	3.0 ± 1.0^ac^	2.2 ± 0.9^ab^	<0.001
95	Social Trends	3.0 ± 0.8	3.0 ± 1.1	3.3 ± 1.2^c^	3.2 ± 1.0^c^	2.6 ± 0.9^ab^	<0.001
105	Tv Exposure	3.6 ± 1.0	3.7 ± 1.2	3.8 ± 1.1^bc^	3.3 ± 1.1^a^	3.1 ± 1.4^a^	0.001
Cluster 5: Social Support and Modelling							
34	Group (Family Peers Partner) PA Behaviours	3.5 ± 0.6	3.1 ± 1.0	4.1 ± 0.9^bc^	3.6 ± 1.0^a^	3.7 ± 1.0^a^	0.010
36	Group Health Habits	3.4 ± 0.8	3.2 ± 1.1	3.7 ± 1.1	3.5 ± 1.0	3.8 ± 0.9	n.s.
62	Parents/Relatives’ Concern About the Environment	2.5 ± 0.7	2.5 ± 0.9	2.9 ± 1.1^bc^	2.0 ± 0.9^a^	2.4 ± 1.1^a^	<0.001
92	Social Expectation	2.9 ± 0.8	2.8 ± 1.1	3.3 ± 1.1^c^	3.1 ± 0.9	2.8 ± 0.9^a^	0.032
93	Social Inclusion	3.1 ± 0.8	2.8 ± 1.1	3.3 ± 1.1	3.1 ± 1.0	3.5 ± 1.1	n.s.
99	Support of Family/Peers/Partner	3.7 ± 0.6	3.3 ± 0.9	4.2 ± 0.9	3.8 ± 0.9	4.0 ± 0.9	n.s.
Cluster 6: Supportive Environment							
2	Access to Personal/Family/Peer Transport	3.2 ± 0.7	3.0 ± 0.9	3.3 ± 1.0	3.1 ± 1.1^c^	3.5 ± 1.0^b^	0.028
43	Involvement in Organized Sport	3.7 ± 0.8	3.7 ± 1.0	4.1 ± 0.9^bc^	3.6 ± 1.0^ac^	3.2 ± 1.2^ab^	<0.001
55	Number of Household Cars/Car Ownership	2.7 ± 0.8	2.6 ± 1.1	2.7 ± 0.9	2.9 ± 1.0	2.6 ± 1.0	n.s.
74	Physician Advices	3.4 ± 0.8	3.5 ± 1.3	2.6 ± 1.0^bc^	3.3 ± 0.9^ac^	4.1 ± 0.8^ab^	<0.001
75	Private Environment (Home/Backyard Space)	3.1 ± 0.9	2.9 ± 1.1	3.3 ± 1.1	3.0 ± 1.0^c^	3.5 ± 1.1^b^	0.023
101	Time Spent Outdoor/Playing Spaces	3.6 ± 0.8	3.7 ± 0.9	4.1 ± 0.9^bc^	3.1 ± 1.1^a^	3.2 ± 1.2^a^	<0.001

Means ± SD of Ratings (Likert scale: 1 = lowest value, 5 = highest value) and Differences (*p* < 0.05) between Population-Level Effect

^a^ = differences (*p* < 0.05) with respect to the youth population

^b^ = differences (*p* < 0.05) with respect to the adult population

^c^ = differences (*p* < 0.05) with respect to the older adult population

n.s. = not significant

### Structuring (sorting and rating)

The recruitment resulted in seventy-nine experts willing to participate in the concept mapping exercise (response rate to invitation was 21 %). While participants affiliated with academic or research institutions (*n* = 64) declared expertise in Life Sciences (78 %; e.g., biology, biochemistry, biotechnology, biomechanics, clinical sciences, developmental and ageing sciences, ergonomics, epidemiology, physiology, medicine, nutrition, neurosciences, public health and health promotion, movement and sport sciences), Social Sciences and Humanities (20 %; e.g., anthropology, behavioural sciences, economics and finance, environmental science, history, law, philosophy, psychology, pedagogy, political science, and sociology), and Physical Sciences and Engineering (2 %; e.g., statistics), those affiliated with European organizations relevant to PA promotion (*n* = 15) declared expertise in Life Sciences (47 %; e.g., movement and sport sciences) and Social Sciences and Humanities (53 %; e.g., economics and finance, law, management, political science, and sociology). The majority of participants (90 %) was from the European Member States (Austria, Belgium, Croatia, Denmark, Finland, Germany, Hungary, Ireland, Italy, Latvia, Poland, Portugal, Slovenia, Spain, Sweden, and United Kingdom), whereas the others (10 %) came from Kosovo, Norway, Russia, Switzerland, and Turkey. Thus, the sample was considered representative of a Pan-European expertise in PA research and promotion.

Respondents were informed about the aim of the investigation and the procedures to access an online analysis platform (i.e., Ariadne; http://www.minds21.biz/). A three-week timeframe was given to complete the clustering and rating of factors. The factors were entered into a project-specific Ariadne software [54], which has been used previously to develop theoretical public health frameworks in Europe [20, 29, 35, 40]. The instructions stated that each factor had to be assigned to one group only, with a maximum number of ten groups permitted. Participants were also required to rate the 106 factors on a Likert-type scale from 1 (lowest value) to 5 (highest value). Ratings were required for factor modifiability across the life course and for the expected population-level effect for youth, adults, and older adults, respectively.

### Analysis and interpretation

The concept mapping software (Ariadne) uses a combination of statistical techniques. First, it computes a binary symmetric similarity matrix per respondent. Second, it provides an aggregated (group) matrix by counting the individual matrices, with high values indicating that many of the participants put the named factors together in a group which implies a conceptual similarity between statements. This aggregated similarity matrix is then used as the input for a (non-metric) principal component analysis (PCA), a technique for translating the distances between statements into coordinates in a multidimensional space. A stepwise analysis from the lowest number (e.g. 2) to the highest number (e.g. 18) of clustering of factors and the graphic representation of their origin are provided. In general, the spatial distribution of the clusters on the map (e.g., eastern, western, northern, and southern parts) mirrors different themes [54].

After the collection of the data from the experts, during a second workshop the DEDIPAC-KH research team analysed the findings to facilitate an agreement for a cohesive EU-PAD framework. A stepwise analysis was performed to provide a configuration of the least number of clusters that possessed reasonable and agreed theoretical distinctions. Through further discussion, members of the DEDIPAC-KH research team determined the labels that would best represent the content of the final configuration of clusters based on their included factors. A consensus on face validity was reached. A priority for research score was estimated for each factor by weighting its grading scores of modifiability (50 %) and the sum of population-level effects (50 %). Priority between clusters was established based on the mean values of their weighted grading. Finally, the proposed EU-PAD framework was submitted online to the participating European experts requesting a final consensus regarding the labelling of the clusters and how the included factors represent research priorities within each cluster. An analysis of variance was performed to test differences (*p* < 0.05) in the level of impact of the 106 factors in the youth, adult, and older adult populations. When significant differences emerged, Bonferroni post-hoc comparisons were used.

According to the literature [21, 33, 37, 39, 55, 56] and to enhance the exploitation of findings for decision making directing future strategic plans, the mean ratings of the modifiability (x-axis) and population-level effect (y-axis) for the youth, adult, and older adult populations were used to plot the position of each factor relative to all other factors. The resulting scatterplots identified four quadrants (e.g., I, II, III, and IV) of “Go-Zones”, reporting factors deemed relevant for their population-level effect but considered to have a low modifiability (Quadrant I), factors that have been attributed low ratings for both modifiability and population-level effect (Quadrant II), factors deemed relevant for their modifiability but considered to have a low population-level effect (Quadrant III), and factors deemed to be most modifiable and having the highest population-level effect (Quadrant IV), respectively. In particular, the Quadrant IV identifıed the factors experts rated as highly important for increasing PA behaviours for each age group.

## Results

### Clustering and ratings of factors

**Fig. 1:**
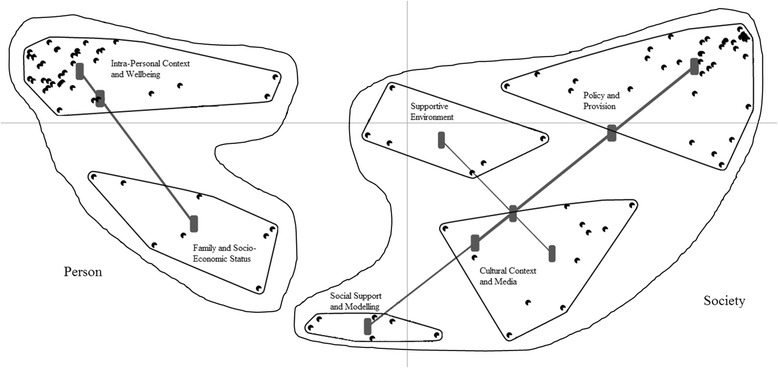
Six-cluster map within the two main areas ‘Person’ and ‘’Society’. Legend; *Straight lines* represent the origin of the clusters from the 3, 4, and 5 cluster arrangements. In particular, the area ‘Person’ originated two clusters (e.g., ‘Intra-Personal Context and Wellbeing’ and ‘Family and Socio-Economic Status’), whilst the area ‘Society’ originated the cluster ‘Policy and Provision’ and a second cluster that, in turn, originated the clusters ‘Cultural Context and Media’, ‘Supportive Environment’, and ‘Social Support and Modelling’

The preliminary analysis of the concept mapping generated two main areas (Fig. 1), the first comprising 42 % of all factors mainly related to the individual (e.g., ‘Person’), the second comprising 58 % of all factors mainly related to socio-cultural-organizational factors (‘Society). Then, the area ‘Person’ generated two clusters, which were labelled based on the analysis of the included factors as Cluster 1 ‘Intra-Personal Context and Wellbeing’ (i.e., encompassing everything related to the individuals including, but not limited, to their health and wellbeing), and Cluster 2 ‘Family and Social Economic Status’ (i.e., referring to the family environment and social status of the individuals). The area ‘Society’ generated four clusters, which were labelled based on the analysis of the included factors as Cluster 3 ‘Policy and Provision’ (i.e., incorporating political aspects that influence the civic life of individuals/groups at local, national, and international levels); Cluster 4 ‘Cultural Context and Media’ (i.e., referring to the cultural and social environment that individuals/groups live in and interact with); Cluster 5 ‘Social Support and Modelling’ (i.e., incorporating factors related to the habits of family/groups that influence the individual); and Cluster 6 ‘Supportive Environment’ (i.e., referring to the factors that influence the engagement in active lifestyles). Final consensus agreement for the cluster labels obtained through an online survey to all participants ranged from 92.7 % (e.g., ‘Family and Social Economic Status’) to 100.0 % (e.g., ‘Social Support and Modelling’).

Table 2 presents the 106 factors organized by cluster, including statistics for ratings of priority for research, modifiability, and population-level effects. The number of factors in each cluster ranged from six in the ‘Supportive Environment’ and ‘Social Support and Modelling’ clusters to 38 in the ‘Policy and Provision’ cluster. For the population-level effect age-related differences (*p* < 0.05) emerged for sixty-seven factors. Post-hoc analysis identified 12 factors that varied across all age groups (e.g., ‘Fear of Injuries/Falling’, ‘Health Status’, ‘Level of Autonomy/Time Management’, ‘Perceived Stress/Life Stressors’, ‘Sub-Pathology/Pathology/Injuries/Pain/Rehabilitation’, and ‘Time Availability’ in the ‘Intra-Personal Context and Wellbeing’ cluster; ‘School/Office Hours’ in the ‘Policy and Provision’ cluster; ‘Cyber Space’, ‘Internet Availability’, and ‘Social Media’ in the ‘Cultural Context and Media’ cluster; ‘Involvement in Organized Sport’ and ‘Physician Advices’ in the ‘Supportive Environment’ cluster). No factors in the other two clusters demonstrated a similar difference across stages of the life course (see Table 2). Post-hoc analysis did not confirm a significant difference only for the factor ‘Actual Body Mass Index’.

### Go-zones

**Fig. 2:**
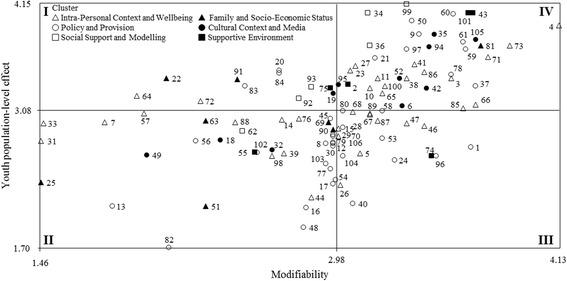
Go-Zone map of modifiability and population-level effect ratings for the youth population

**Fig. 3:**
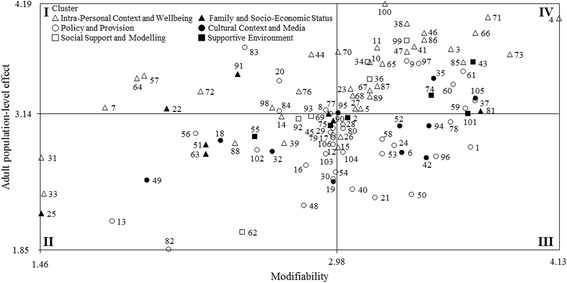
Go-Zone map of modifiability and population-level effect ratings for the adult population

**Fig. 4:**
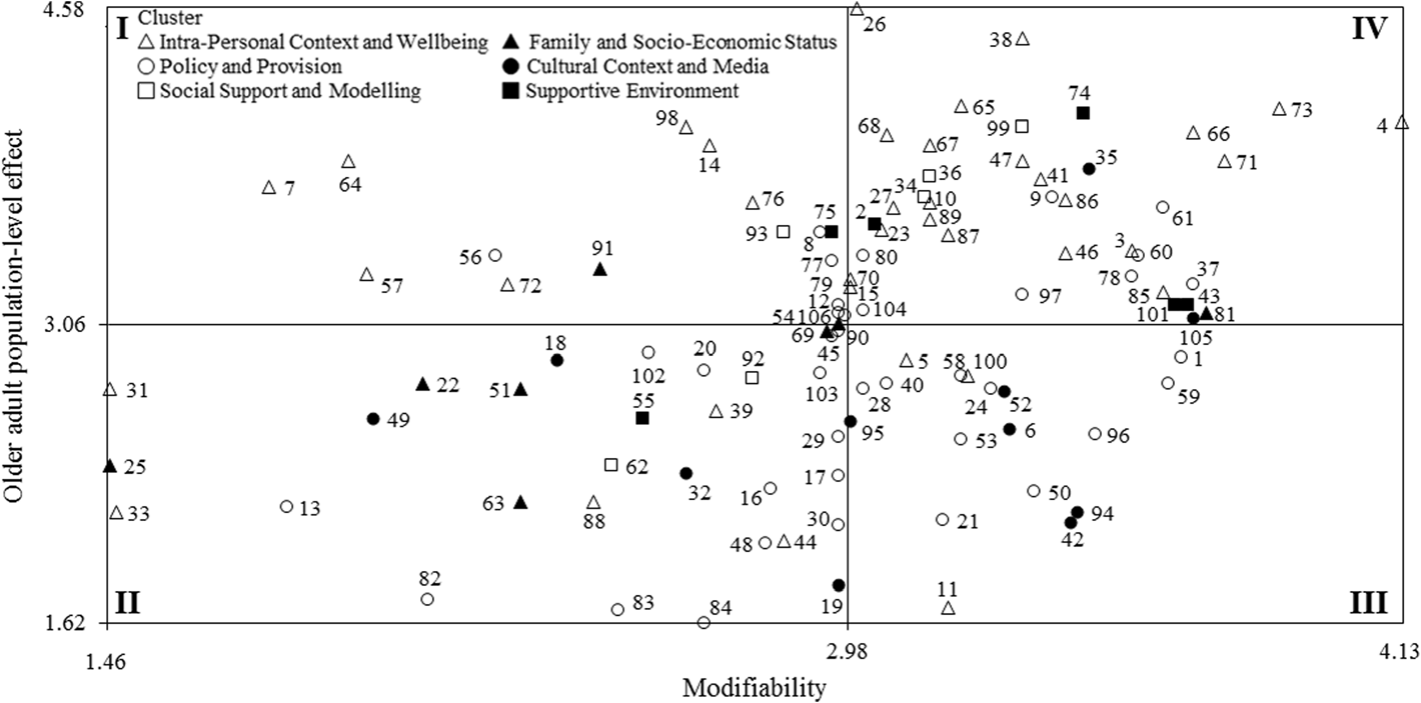
Go-Zone map of modifiability and population-level effect ratings for the older adult population

The relationships between modifiability and population-level effect for the youth, adult, and older adult populations are presented in Figs. 2, 3 and 4, respectively. Factors receiving high ratings for both modifiability and level effect are presented in Quadrant IV, in which 45 factors were noted for both the youth and older adult populations, and 47 factors for the adult population. Twenty-five of these factors were common between the three age populations (Table 3). The majority of these factors belonged to the ‘Intra-Personal Context and Wellbeing’ and the ‘Policy and Provision’ clusters, representing 52 and 20 % of the total, respectively. The rest belonged to the clusters ‘Social Support and Modelling’, ‘Cultural Context and Media’, ‘Supportive Environment’, and ‘Family and Social Economic Status’, representing 12, 8, 4 and 4 % of the total, respectively.

**Table 3:** Factors by clusters included in the quadrant IV of Go-Zones (youth, adults, and older adults)

Cluster	Factor Number and Statement
Intra-Personal Context and Wellbeing	3 Actual Body Mass Index
	4 Actual PA Level
	10 Beliefs/Values
	23 Emotions
	27 Feeling of Inadequacy (Too Clumsy/Too Old)/Teasing
	38 Health Status
	41 Intentions/Attitudes
	65 Perceived Barriers
	66 Perceived Benefits of PA
	71 Personal Goals/Outcome Expectancies/Atchievement Orientation/Motivation
	73 Physical fitness levels (Strength, Endurance, Coordination, Agility, Flexibility)
	85 Self PA Monitoring
	86 Self Perceptions (Awareness, Confidence, Efficacy, Body Image, PA Level)
Family and Socio-Economic Status	81 Rewards (Encouragement/Support)
Policy and Provision	9 Availability/Access/Proximity of PA Organized Sport Facilities Tools
	37 Health Education
	60 PA Programs in School/Office/Community
	61 PA Programs/Plans
	97 Sports Facilities
Cultural Context and Media	35 Group Activity (Outdoor/Indoor)
	105 Tv Exposure
Social Support and Modelling	34 Group (Family Peers Partner) PA Behaviours
	36 Group Health Habits
	99 Support of Family/Peers/Partner
Supportive Environment	43 Involvement in Organized Sport

In Quadrant IV, five factors were present only for the youth population (factors ‘Mandatory PA in Community/Schools’ and ‘PA and Sport Organizations Advocacy’ in cluster ‘Policy and Provision’, and factors’Advertisement’,’Cyber Space’, and ‘Internet Availability’ in cluster ‘Cultural Context and Media’) and five factors only for the older adult population (factors ‘Conscious Control of Automated Body Movements’ and ‘Fear of Injuries/Falling’ in cluster Intra-Personal Context and Wellbeing’, and factors’Mobility Policy’, ‘Public Transport Policies’, and ‘Transport Policies’ in cluster ‘Policy and Provision’). Two factors were present only in the adult population (factor ‘Addictions (Smoking Gambling Drugs)’ in cluster ‘Intra-Personal Context and Wellbeing’, and factor ‘Financial Measures and Regulation for PA and Sport’ in cluster ‘Policy and Provision’). Five factors were shared only between the youth and adult populations (factors ‘Capability to Combine Sport and Education/Work Requirements (Dual Career)’ and ‘Time Availability’ in cluster ‘Intra-Personal Context and Wellbeing’, and factors’Media’,’Social Media’, and ‘Social Trends’ in ‘Cultural Context and Media’), whereas six factors were shared only between adult and older adult populations (factors’Level of Autonomy/Time Management’, ‘Life Satisfaction’, and ‘Perceived Stress/Life Stressors’ in cluster ‘Intra-Personal Context and Wellbeing’, factor ‘Social Competence/Role’ in cluster Family and Social Economic Status’, factor ‘City Planning’ in cluster Policy and Provision’, and factor ‘Physician Advices’ in cluster ‘Supportive Environment’).

### Priority for research

Table 4 presents the top five factors based on mean rating for the priority for research for each cluster. The overall mean rating ranged from 3.4 ± 0.2 for the cluster ‘Supportive Environment’ to 2.7 ± 0.5 for the cluster ‘Family and Socio-Economic Status’. In particular, the most prioritised factors for research were ‘Actual PA Level’, ‘Physical Fitness Levels (Strength, Endurance, Coordination, Agility, Flexibility)’, and ‘Personal Goals/Outcome Expectancies/Achievement Orientation/Motivation’, all belonging to the cluster ‘Intra-Personal Context and Wellbeing’. Final consensus agreement for the priority of research of factors within each cluster obtained through an online survey to all participants ranged from 83 % (e.g., ‘Intra-Personal Context and Wellbeing’) to 98 % (e.g., ‘Social Support and Modelling’).

**Table 4:** Highest-rated factors for priority for research in descending order for each cluster

Factor #	Statement By Cluster	Priority for Research
	Cluster 1: Intra-Personal Context and Wellbeing	3.2 ± 0.5
4^a^	Actual PA Level	4.1 ± 0.6
73^a^	Physical Fitness Levels (Strength, Endurance, Coordination, Agility, Flexibility)	3.9 ± 0.7
71^a^	Personal Goals/Outcome Expectancies/Achievement Orientation/Motivation	3.8 ± 0.7
66^a^	Perceived Benefits of PA	3.7 ± 0.7
38^a^	Health Status	3.7 ± 0.7
	Cluster 2: Family and Socio-Economic Status	2.7 ± 0.5
81^a^	Rewards (Encouragement/Support)	3.5 ± 0.8
69	Perceived Social Role	3.0 ± 0.8
90	Social Competence/Role	3.0 ± 0.8
91	Social Economic Status/Personal Income (for Children: Parents’ Income)/Level of Education	2.9 ± 0.8
22	Educational Level (Parents/Relatives)	2.6 ± 0.8
	Cluster 3: Policy and Provision	3.0 ± 0.4
61^a^	PA Programs/Plans	3.6 ± 0.8
60^a^	PA Programs in School/Office/Community	3.6 ± 0.8
9*	Availability/Access/Proximity of PA Organized Sport Facilities/Tools	3.6 ± 0.7
37^a^	Health Education	3.5 ± 0.7
59	PA Education (at School/Work)/Knowledge of Effects of PA	3.4 ± 0.9
	Cluster 4: Cultural Context and Media	3.0 ± 0.4
35^a^	Group Activities (Outdoor/Indoor)	3.6 ± 0.7
105^a^	TV Exposure	3.6 ± 1.0
94	Social Media	3.2 ± 0.9
52	Media	3.2 ± 0.8
42	Internet Availability	3.1 ± 0.9
	Cluster 5: Social Support and Modelling	3.3 ± 0.3
99^a^	Support of Family/Peers/Partner	3.7 ± 0.6
34^a^	Group (Family/Peers/Partner) PA Behaviours	3.5 ± 0.6
36^a^	Group Health Habits	3.4 ± 0.8
93	Social Inclusion	3.1 ± 0.8
92	Social Expectations	2.9 ± 0.8
	Cluster 6: Supportive Environment	3.4 ± 0.2
43^a^	Involvement in Organized Sport	3.7 ± 0.8
101	Time Spent Outdoor/Playing Spaces	3.6 ± 0.8
74	Physical Advices	3.4 ± 0.8
2	Access to Personal/Family/Peer Transport	3.2 ± 0.7
75	Private Environment (Home/Backyard Space)	3.1 ± 0.9

^a^Indicates inclusion also in the Quadrant IV of the youth, adult, and older adult populations

## Discussion

The current DEDIPAC-KH initiative aimed to develop the EU-PAD framework to provide a Pan-European and life course view of key factors of PA behaviours, and a proposal of how these factors may group into clusters. The combined experience of European scholars and policy makers was capitalized to identify potential factors of PA behaviours and to rate these factors in terms of their importance (level of effect) to the three populations of concern (e.g., youth, adults, and older adults), their modifiability and their priority for research. Not only is the response to these three questions indicative of our current understanding of the determinants of PA behaviours, it could also provide significant guidance to the future research agenda within Europe and a structure to increase collaboration and harmonisation of research methodologies. In fact, the identification of the six clusters fits well into the theoretical perspective of research utilization, which is considered as an important condition for implementing research findings and subsequent translation into policies [20].

The EU-PAD framework presents a conceptual map to generate recommendations but not conclusions and the findings are relevant to all who have contributed [22]. The uniqueness of the present study lies in the effort in synergising a range of knowledge, capacities, activities, and actions of multiple European experts in an attempt to uncover the multi-level relationships between PA factors applicable to individuals and to society. As expected, experts in the European Research Area of Physical Sciences and Engineering resulted underrepresented compared to those with an expertise in Life Sciences and Social Sciences and Humanities who are more likely to be involved in PA research and European PA organizations. Considering the broad and pervasive nature of PA, the European research agenda should foster research for the promotion of PA also in the disciplines that are typically less associated with PA. The practical relevance of the EU-PAD framework subsumes the resulting theoretical evidence as a sound basis for practical decision-making [20], and urges policy makers and scholars from different disciplines to coordinate their efforts in bridging existing gaps between sciences, practices, and policies in the HEPA area [57]. Thus, the results of this initiative could potentially contribute to the development of a strategic plan for both research and policies at a Pan-European level, and ultimately to more effective European policies and actions in promoting positive PA behaviours [19].

To yield the optimal practical outcomes either at the society level or at the person level, it is critical to consider the importance (level of effect) of each factor to the three populations of interest (i.e., youth, adults, and older adults) and the level of modifiability across the life course. Of the 25 factors identified to be the most modifiable and influential across the life course, 16 of them were rated to be amongst the top 5 of research priority list in each cluster (see Table 4). In all, research focus on these 16 factors might result in a more targeted and fruitful approach for promoting positive PA behaviours across the life course. Despite the high consensus for the priority of research of factors within each cluster (83–98 %), this prioritisation of discrete factors could be less valuable than the identification of discrete or group of factors for specific life stages or contexts [41]. Furthermore, emphasis should be placed on transdisciplinary investigations and interventions, in line with the aim of the European Joint Programming Initiative A Healthy Diet for a Healthy Life to foster a common research agenda for the enhancement of active lifestyles of European citizens [18]. In fact, researchers or policy makers ought not lose sight of the other PA factors because all operate within their respective clusters and interactions between them are complex and ultimately, a holistic view to interpreting the clusters in the framework is needed. Moreover, particular attention should be given to relatively modifiable factors that are unique to a specific age group (e.g., for youth: ‘Cyber Space’; for adults: ‘Financial and Regulation for PA and Sport’; and for older adults: ‘Mobility Policy’) as they might influence PA behaviours for each population in a distinctive way [7, 58, 59].

In extending the examination of the factors in distinct clusters, the analysis noted two core themes from the six clusters within the framework: 1) the ‘Person’ (referring to the proximal relationships between individuals such as family, social relationships, and socioeconomic status), and 2) the ‘Society’ (encompassing environmental, historical, political, social, economic, scientific, cultural, and organization factors), each comprising a cluster with around 35 % of all the factors (e.g., ‘Intra-Personal Context and Wellbeing’ and ‘Policy and Provision’, respectively). Resonating previous research and systematic literature reviews on the determinants of PA, the ‘Person’ theme infers that the individual is central in the adoption of an active lifestyle, including, but not limited to, individual responsibility, personal committment and lifestyle choices for PA behaviours [13, 16, 17, 60–65], whilst the ‘Society’ theme echoes researchers’ plead for attention to the role of policy and the environment in promoting PA in European citizens [13, 16, 17, 66]. Interestingly, out of all the clusters, ‘Supportive Environment’ was considered to be the highest priority for research. This might suggest, and call for, a shift in focus from individual responsibility, personal commitment, and lifestyle choices to influences of supportive environments for overcoming barriers to PA for different age groups at both research and policy levels. Furthermore, the highest ranked factor in this cluster, ‘Involvement in Organized Sports’, is closely related to those positioned at the highest level in clusters within the ‘Person’ theme (e.g., ‘Actual PA Level’,’Rewards (Encouragement/Support)’, and ‘Support of Family/Peers/Partner’). This finding substantiates the complexity and interrelatedness of all the factors in this EU-PAD framework and the personal and social relevance of organized sports for sustainable collaboration programmes to increase active lifestyles. This finding is in line with the recommendations of the European Expert Group on HEPA, which urge Governments to facilitate population level behavioural change by creating supporting and enabling environments for an active lifestyle [4].

According to Trochim and colleagues [34], concept mapping has been considered a cost-effective and successful way of identifying factors determining PA behaviours, despite this inherent value it must be accepted that the process involves very demanding and time-intensive activities, such as brainstorming, the management of a large amount of information, the complex scenery of interrelated ideas to be systematised, and the recruitment of European experts from a diversity of disciplines for reaching a consensus. Some limitations to the present study have been noted. First and foremost, the selection bias of the diverse group of stakeholders engaged in this study cannot be ruled out. Secondly, despite the initial intent to provide a more exhaustive picture of potential factors influencing PA behaviours, experts’ prior knowledge in the field of PA research would almost inevitably influence the list of factors to be included in the current study. When repeating the concept mapping exercise again, it could be advisable to engage additional experts from fields of research that are distinct from the current focus (e.g., urban planning disciplines), to operationalize and measure factors separately within specific areas of competence, and to combine them through a collective effort of inter- and trans-disciplinary expertise to enrich our understanding of PA determinants and their interactions (such as the mediating, moderating and causal role of each determinant to PA behaviours). It is unknown what influence the inclusion of experts from more diverse fields, albeit PA-related, may have on the outcome of the concept mapping exercise.

## Conclusions

In conclusion, the cumulated experience and perception of European scholars from different scientific areas and policy makers in the field of sport and HEPA were integrated into a framework of factor clusters which both illuminates and confirms the complexity of PA behaviours phenomenon. In fact, the EU-PAD framework identifies the importance of addressing multiple factors within and between clusters. The factors and clusters present some similarities with respect to those described by previous ecological models for understanding determinants of PA behaviours [13, 16, 17]. Distinctly, by using a concept mapping approach the EU-PAD framework has provided additional and new insights regarding a European and life course view of key factors, additional definition regarding the specific nature of the factors and how these factors group into clusters. In practice, the EU-PAD framework can be used to (i) guide the development of a strategic plan for novel and multi-disciplinary research at Pan-European level addressing the complexity of determinants of PA behaviours across the life course (e.g., evidence production); (ii) identify key aspects for potential strategies to implement multi-sectoral European policies in HEPA (e.g., agenda setting and advocacy); and (iii) develop intervention programmes for individual behavioural change and interventions for impinging on the social and physical (natural and built) environment to improve the involvement of European citizens in healthy active lifestyles (e.g., HEPA guidelines adoption, implementation and delivery). To fully exploit effective actions to increase PA levels, the well-established European platforms (e.g., the Regional Office for Europe of the World Health Organization, the European Sport Forums, the meetings of sports Directors, sport and education Ministers, and Expert Groups, the conferences of the Council Presidency, and of the Enlarged Partial Agreement on Sport of the Council of Europe) provide a valuable infrastructure to enhance communication and cooperation between relevant stakeholders at national and European levels for the development and implementation of an integrated approach to healthy active lifestyle interventions. In fact, future European research and intervention plans are still needed to verify specific mechanisms through which particular influences may interact and implement active lifestyle behaviours of European citizens.

## Abbreviations

DEDIPAC-KH: DEterminants of DIet and Physical ACtivity Knowledge Hub
EU: European Union
EU-PAD: EUropean-Physical Activity Determinants
HEPA: Health-enhancing physical activity
PA: Physical activity
PCA: Principal component analysis
